# Ethical Challenges in the Use of Digital Tools for Screening of Depression in India: A Scoping Review

**DOI:** 10.1192/bjo.2024.262

**Published:** 2024-08-01

**Authors:** Francesca Townsend, Ahmed Waqas, Atif Rahman

**Affiliations:** 1 University of Liverpool, Liverpool, United Kingdom

## Abstract

**Aims:**

Depression poses a significant public health concern globally, characterized by prolonged periods of sadness, loss of interest, and impairment in daily functioning. With over 800,000 annual deaths attributed to suicide, it stands as the second leading cause of mortality among 15–29-year-olds worldwide. To address this growing crisis, various digital methods are being increasingly developed for screening depression efficiently in large populations. However, the ethical implications surrounding the use of these tools remain debated. This scoping review aims to explore the landscape of research on digital screening methods for depression in India, elucidating ethical challenges and identifying research gaps.

By synthesizing available evidence, this study seeks to contribute to the discourse surrounding the ethical use of digital tools for depression screening in India, ultimately striving for improved mental health outcomes in the population.

**Methods:**

Using a pre-tested search strategy in January 2024, we searched PubMed and Google Scholar for studies regarding digital divide in the use of digital technology for mental health. Relevant studies were selected using a two phased screening process. Studies included in the review were synthesised qualitatively using a thematic synthesis approach.

**Results:**

Out of 379 titles identified in our database search, only four were included in the qualitative synthesis. Two of these were cross-sectional, followed by a qualitative study and a pre-post evaluation. These studies were conducted in remote villages in the state of Andhra Pradesh, urban slums of Delhi, pan-Indian national survey and rural and under resourced urban areas.

The studies examined diverse aspects of the digital divide in India, revealing profound socio-economic disparities and gender inequities. Disparities in ownership of digital devices and usage were stark, with less educated, lower-income, and lower-caste groups facing marginalization due to limited access and skills. There were gender discrepancies in mobile phone ownership and internet access, with females significantly less likely to possess these technologies compared with males. However, there is a strong potential of mobile technology in increasing mental health service utilization in rural areas, fostering community awareness and stigma reduction.

**Conclusion:**

Collectively, these findings illuminate the multifaceted challenges of the digital divide in India, emphasizing the urgent need for targeted interventions to promote equitable access to technology and bridge socio-economic gaps.

